# Ability of the Met Kinase Inhibitor Crizotinib and New Generation EGFR Inhibitors to Overcome Resistance to EGFR Inhibitors

**DOI:** 10.1371/journal.pone.0084700

**Published:** 2013-12-26

**Authors:** Shigeki Nanjo, Tadaaki Yamada, Hiroshi Nishihara, Shinji Takeuchi, Takako Sano, Takayuki Nakagawa, Daisuke Ishikawa, Lu Zhao, Hiromichi Ebi, Kazuo Yasumoto, Kunio Matsumoto, Seiji Yano

**Affiliations:** 1 Division of Medical Oncology, Cancer Research Institute, Kanazawa University, Kanazawa, Japan; 2 Laboratory of Translational Pathology, Hokkaido University Graduate School of Medicine, Sapporo, Japan; 3 Division of Tumor Dynamics and Regulation, Cancer Research Institute, Kanazawa University, Kanazawa, Japan; Case Western Reserve University, United States of America

## Abstract

**Purpose:**

Although EGF receptor tyrosine kinase inhibitors (EGFR-TKI) have shown dramatic effects against EGFR mutant lung cancer, patients ultimately develop resistance by multiple mechanisms. We therefore assessed the ability of combined treatment with the Met inhibitor crizotinib and new generation EGFR-TKIs to overcome resistance to first-generation EGFR-TKIs.

**Experimental Design:**

Lung cancer cell lines made resistant to EGFR-TKIs by the gatekeeper *EGFR*-T790M mutation, *Met* amplification, and HGF overexpression and mice with tumors induced by these cells were treated with crizotinib and a new generation EGFR-TKI.

**Results:**

The new generation EGFR-TKI inhibited the growth of lung cancer cells containing the gatekeeper *EGFR*-T790M mutation, but did not inhibit the growth of cells with *Met* amplification or HGF overexpression. In contrast, combined therapy with crizotinib plus afatinib or WZ4002 was effective against all three types of cells, inhibiting EGFR and Met phosphorylation and their downstream molecules. Crizotinib combined with afatinib or WZ4002 potently inhibited the growth of mouse tumors induced by these lung cancer cell lines. However, the combination of high dose crizotinib and afatinib, but not WZ4002, triggered severe adverse events.

**Conclusions:**

Our results suggest that the dual blockade of mutant EGFR and Met by crizotinib and a new generation EGFR-TKI may be promising for overcoming resistance to reversible EGFR-TKIs but careful assessment is warranted clinically.

## Introduction

Lung cancers with mutations that activate epidermal growth factor receptor (EGFR), including exon 19 deletions and the exon 21 L858R point mutation, respond to the reversible EGFR-tyrosine kinase inhibitors (EGFR-TKIs) gefitinib and erlotinib [1]. These mutations have been shown to promote the activation of EGFR signaling and tumor dependency on EGFR. Recent clinical trials have shown that progression-free survival (PFS) in patients with EGFR mutant lung cancer is prolonged by treatment with a reversible EGFR-TKI and the irreversible EGFR-TKI afatinib, which was designed to covalently bind to EGFR [2-5]. Nevertheless, almost all responders relapse after acquiring resistance to these EGFR-TKIs [1,6]. Among the mechanisms by which cancer cells become resistant to reversible EGFR-TKIs are 1) gatekeeper mutations in *EGFR*, such as the T790M second mutation [7,8]; 2) activation of bypass signaling caused by *Met* amplification [9], hepatocyte growth factor (HGF) overexpression [10], or Gas6-Axl activation [11]; 3) activation of downstream molecules (PTEN loss or *PIK3CA* mutation) [12,13]; 4) small-cell lung cancer transformation [14]; and 5) epithelial-to-mesenchymal transition [15]. The gatekeeper *EGFR*-T790M mutation is the most frequent, occurring in half of tumors with acquired resistance to reversible EGFR-TKIs. The methionine residue at position 790 generates a bulkier side chain, which enhances the affinity of the EGFR tyrosine kinase pocket with ATP [7], decreasing the effective binding of gefitinib and erlotinib to the tyrosine kinase pocket of EGFR [16]. To overcome acquired resistance, a new generation of EGFR-TKIs was developed, including irreversible EGFR-TKIs and mutant-selective EGFR-TKIs [17-22]. In clinical trials, however, several of these irreversible EGFR-TKIs failed to meet their primary endpoints in EGFR-TKI-refractory lung cancer and induced severe toxicities, such as diarrhea, skin rash/acne, stomatitis, and nail effects [1,20]. 

HGF, the sole ligand of Met, is important in the development of EGFR-TKI resistance in *EGFR* mutant lung cancer cells. HGF activates Met phosphorylation and stimulates the downstream Akt and Erk1/2 pathways utilizing Gab1, an adaptor protein for Met, triggering resistance to both reversible and irreversible EGFR-TKIs [10,23,24]. In our previous Japanese cohort study of patients with *EGFR* mutant lung cancer, high HGF expression was detected in 61% of tumors with acquired resistance and in 29% of tumors with intrinsic resistance to EGFR-TKIs, suggesting that targeting HGF may overcome resistance to EGFR-TKIs [25]. 

Resistance to molecular targeting agents may be caused by tumor heterogeneity. For example, we and other researchers reported that HGF overexpression can exist together with gatekeeper *EGFR*-T790M mutation or *Met* gene amplification in EGFR mutant lung cancer with acquired resistance to EGFR-TKIs [23,25]. Therefore, HGF-Met axis signaling can allow tumors to bypass the effects of new generation EGFR-TKIs. Agents that overcome resistance to EGFR inhibitors, especially by HGF-Met bypass signaling, are urgently needed. Crizotinib is a dual tyrosine kinase inhibitor of ALK and Met that shows potent anti-tumor activity, safety and feasibility as monotherapy in lung cancer patients with *EML4-ALK* rearrangements [26]. We have therefore evaluated the efficacy and feasibility of combinations of crizotinib and new generation EGFR inhibitors in overcoming the resistance to EGFR-TKIs of lung cancer cells harboring *EGFR* mutations. 

## Materials and Methods

### Cell cultures and reagents

The *EGFR* mutant human lung adenocarcinoma cell lines PC-9 (del E746_A750) and HCC827, with deletions in *EGFR* exon 19, were purchased from Immuno-Biological Laboratories Co. (Takasaki, Gunma, Japan) and the American Type Culture Collection (Manassas, VA), respectively. HCC827ER cells, with deletions in *EGFR* exon 19 and *Met* gene amplification [27], and H1975 cells, with the L858R/T790M double mutation in *EGFR* [28], were kindly provided by Drs. Kenichi Suda and Tetsuya Mitsudomi (Aichi Cancer Center Hospital, Nagoya, Japan), and by Drs. Yoshitaka Sekido (Aichi Cancer Center Research Institute, Japan) and John D. Minna (University of Texas Southwestern Medical Center), respectively. PC-9/KGR1, with deletions in *EGFR* exon 19 and the T790M double mutation (Table **1**), were established from PC-9 cells after the stepwise exposure to gefitinib in 2011 at Kanazawa University (Kanazawa, Japan). These cell lines were maintained in RPMI 1640 medium supplemented with 10% fetal bovine serum (FBS), penicillin (100 U/mL), and streptomycin (50 g/mL), in a humidified CO2 incubator at 37°C. The MRC-5 lung embryonic fibroblast cell line was obtained from RIKEN Cell Bank. MRC-5 (P 25–30) cells were cultured in Dulbecco’s Modified Eagle Medium (DMEM) supplemented with 10% FBS, penicillin (100 U/mL), and streptomycin (50 g/mL), in a humidified CO2 incubator at 37°C.

**Table 1:** Status of EGFR mutation and Met amplification and HGF expression in the culture medium of each cell line.

Cell line	EGFR mutation status	*Met* amplification	HGF production (ng/10^5^cells/24hr)
HCC827	E746_A750del	-	<0.03
HCC827ER	E746_A750del	+	<0.03
HCC827/Vec	E746_A750del	-	<0.03
HCC827/HGF#24	E746_A750del	-	284±29
HCC827/HGF#28	E746_A750del	-	121±21
PC-9	E746_A750del	-	<0.03
PC-9 KGR1	E746_A750del/T790M	-	<0.03
H1975	L858R/T790M	-	<0.03
H1975/Vec	L858R/T790M	-	<0.03
H1975/HGF	L858R/T790M	-	370±24
MRC-5	wild	-	88±9.0

The characteristics of these cell lines are summarized in Table **1**. All cells were passaged for less than 3 months before renewal from frozen, early-passage stocks. Cells were regularly screened for mycoplasma, using MycoAlert Mycoplasma Detection Kits (Lonza, Rockland, ME). Crizotinib, afatinib, and WZ4002 were obtained from Selleck Chemicals (Houston, TX). Erlotinib was obtained from Chugai Pharmaceutical Co. Ltd. (Tokyo, Japan). Human recombinant HGF was prepared as described [29]. 

### *HGF* gene transfection

One day before transfection, aliquots of 1×10^5^ HCC827 and H1975 cells in 1 ml of antibiotic-free medium were plated on 6-well plates. Full-length *HGF* cDNA cloned into the BCMGSneo expression vector [30] was transfected using Lipofectamine 2000 in accordance with the manufacturer’s instructions. After 24 h, the cells were washed with PBS and incubated for an additional 72 h in antibiotic-containing medium, followed by selection in G418 sulfate (Calbiochem, La Jolla, CA). After limiting dilution, HGF-producing cells, HCC827/HGF#24, HCC827/HGF#28, and H1975/HGF, and vector control (HCC827/Vec and H1975/Vec) were established. HGF production by HCC827/HGF#24, HCC827/HGF#28, and H1975/HGF cells was confirmed by ELISA.

### Cell growth assay

Cell proliferation was measured by the MTT dye reduction method [31]. Tumor cells, plated at 2 × 10^3^/100 μL RPMI 1640 plus 10% FBS per well in 96-well plates, were incubated for 24 hours; afatinib, WZ4002, and crizotinib, and/or HGF were added to each well, and incubation was continued for a further 72 hours. Cell growth was measured with MTT solution (2 mg/mL; Sigma, St. Louis, MO), as described [10]. Each experiment was performed at least 3 times, each with triplicate samples. 

### Antibodies and western blotting

Protein aliquots of 25 μg each were resolved by SDS polyacrylamide gel (Bio-Rad, Hercules, CA) electrophoresis and transferred to polyvinylidene difluoride membranes (Bio-Rad). After washing 3 times, the membranes were incubated with Blocking One (Nacalai Tesque, Inc., Kyoto, Japan) for 1 hour at room temperature and then incubated overnight at 4°C with primary antibodies to Met (25H2), phospho-Met (anti-p-Met, Y1234/Y1235; 3D7), p-EGFR (Y1068), Akt, p-Akt (S473) (Cell Signaling Technology, Beverly, MA); human EGFR (1 μg/mL), human/mouse/rat ERK1/ERK2 (0.2 μg/mL), and p-ERK1/ERK2 (T202/Y204; 0.1 μg/mL) (R&D Systems, Minneapolis, MN). After washing thrice, the membranes were incubated for 1 hour at room temperature with species-specific horseradish peroxidase–conjugated secondary antibodies. Immunoreactive bands were visualized with SuperSignal West Dura Extended Duration Substrate, an enhanced chemiluminescent substrate (Pierce Biotechnology, Rockford, IL). Each experiment was performed at least thrice independently. 

### Co-culture of lung cancer cells with fibroblasts

Cells were co-cultured in Transwell Collagen-Coated chambers separated by an 8-μm pore sized filter (BD Biosciences, Erembodegem, Belgium). MRC-5 (1 × 10^4^ cells/300 μL) cells were placed in the upper chamber and incubated in the presence or absence of anti-human HGF antibody (5 μg/mL) or crizotinib (100 nmol/L) for 2 hours. Tumor cells (8 × 10^3^ cells/800 μL), with or without afatinib (100 nmol/L) or WZ4002 (100 nmol/L) were placed in the lower chamber and co-cultured with the MRC-5 cells (1 × 10^4^ cells/300 μL) in the upper chamber for 72 hours. The upper chamber was then removed, 200 µL of MTT solution (2 mg/mL; Sigma) were added to each well and the cells were incubated for 2 hours at 37°C. The media were removed and the dark blue crystals in each well were dissolved in 400 μL of DMSO. Absorbance was measured with an MTP-120 microplate reader (Corona Electric) at test and reference wavelengths of 550 nm and 630 nm, respectively. The percentage growth was measured relative to untreated controls. All samples were assayed at least in triplicate, with each experiment performed three times independently.

### Subcutaneous xenograft models

Suspensions of H1975/Vec and H1975/HGF cells (5×10^6^) were injected subcutaneously into the backs of 5-week-old female severe combined immunodeficiency (SCID) mice (Clea, Tokyo, Japan). After 6 days, the mice were randomized to (a) no treatment (control group), (b) oral afatinib (25 mg/kg/daily), (c) oral WZ4002 (25 mg/kg/daily), (d) low dose oral crizotinib (10 mg/kg/daily), (e) high dose oral crizotinib (25 mg/kg/daily), (f) afatinib plus low dose crizotinib, (g) afatinib plus high dose crizotinib, (h) WZ4002 plus low dose crizotinib, and (i) WZ4002 plus high dose crizotinib. Tumor size and mouse body weight were measured twice per week, and tumor volume was calculated, in mm^3^, as width^2^ × length/2. This study was carried out in strict accordance with the recommendations in the Guide for the Care and Use of Laboratory Animals of the Ministry of Education, Culture, Sports, Science and Technology in Japan. The protocol was approved by the Committee on the Ethics of Experimental Animals, Advanced Science Research Center, Kanazawa University, Kanazawa, Japan (approval no. AP-132618). All surgery was performed under sodium pentobarbital anesthesia, and all efforts were made to minimize suffering. 

### Histological analyses of tumors

Apoptotic cells were detected by terminal deoxynucleotidyl transferase–mediated nick end labeling, using the DeadEnd^TM^ Fluorometric TUNEL system (Promega, Madison, WI). Briefly, formalin fixed, paraffin embedded tissue sections (4 μm thick) were deparaffinized and tissues were permeabilized with protease K solution. The samples were equilibrated, and DNA strand breaks were labeled with fluorescein-12-dUTP (fluorescein-12-2-deoxy-uridine-5-triphosphate) by adding nucleotide mixture and TUNEL. The reaction was stopped by the addition of saline sodium citrate, and the localized green fluorescence of apoptotic cells was detected by fluorescence microscopy (×400). 

Proliferating cells were detected by incubating tissue sections with Ki-67 antibody (Clone MIB-1; DAKO Corp, Glostrup, Denmark). Antigen was retrieved by microwaving tissue sections in 10 mM citrate buffer (pH 6.0). After incubation with secondary antibody and treatment with the Vectastain ABC Kit (Vector Laboratories, Burlingame, CA), peroxidase activity was visualized using the DAB reaction. The sections were counterstained with hematoxylin.

### Quantification of immunohistochemistry and immunofluorescence results

The five areas containing the highest numbers of stained cells within each section were selected for histologic quantitation by light or fluorescent microscopy at 400-fold magnification. All results were independently evaluated by two investigators (S.N. and T.Y.). 

### Histological analysis and quantification of intestinal mucosal damage

Sections were stained with hematoxylin and eosin for routine histologic examinations. Mucosal damage was scored as; normal (0), mild mucosal atrophy (1), moderate mucosal atrophy (2), and severe mucosal atrophy and/or ulcer formation (3) in both the small and large intestines. The two areas within a section in each group of 5 mice were histologically quantitated by microscopy at 400-fold magnification, with scores ranging 0 to 12. All results were independently evaluated by two investigators (H.N. and S.N.).

### RNA interference

Duplexed Stealth RNAi (Invitrogen) against *Met* and *ALK*, and Stealth RNAi Negative Control Low GC Duplex #3 (Invitrogen) were used for RNA interference (RNAi) assays. Briefly, aliquots of 1 × 10^5^ cells in 2 mL of antibiotic-free medium were plated into each well of a 6-well plate and incubated at 37°C for 24 hours. The cells were transfected with siRNA (250 pmol) or scrambled RNA using Lipofectamine 2000 (5 μL) in accordance with the manufacturer’s instructions (Invitrogen). After 24 hours, the cells were washed twice with PBS and incubated in the presence or absence of afatinib (100 nmol/L) or HGF (50 ng/mL) for an additional 48 hours in antibiotic-containing medium. These tumor cells were then used for cell proliferation assays, with *Met and ALK* knockdowns confirmed by western blotting. The siRNA target sequences were *5'-UCCAGAAGAUCAGUUUCCUAAUUCA-3'* and *5'-UGAAUUAGGAAACUGAUCUUCUGGA-3'* for Met and 5'-UCAUUAUCCGGUAUACAGGCCCAGG-3' and 5'- CCUGGGCCUGUAUACCGGAUAAUGA-3' for *ALK*. Each assay was performed using triplicate samples, with three independent experiments performed.

### HGF production in cell culture supernatants

Cells (1 x 10^5^) were cultured in 2 mL of RPMI 1640 or DMEM with 10% FBS for 24 hours, washed with PBS and incubated for 48 hours in RPMI 1640 with 10% FBS. The culture media were harvested and centrifuged, and the supernatants were stored at –70°C until analysis. HGF was measured by ELISA (Immunis HGF EIA; B-Bridge International, Mountain View, CA; limit of detection, 0.1 ng/mL), according to the manufacturer’s instructions. All samples were assayed in triplicate. Color intensity at 450 nm was measured with a spectrophotometric plate reader. Growth factor concentrations were determined by comparison with standard curves. 

### Statistical analysis

The statistical significance of differences was analyzed by one-way ANOVA performed with GraphPad Prism Ver. 4.01 (GraphPad Software, Inc., San Diego, CA, USA). In all analyses, P < 0.05 was defined as statistically significant.

## Results

### Crizotinib and a new generation EGFR-TKI overcomes resistance to new generation EGFR-TKI in lung cancer cells harboring EGFR mutations

**Figure 1:**
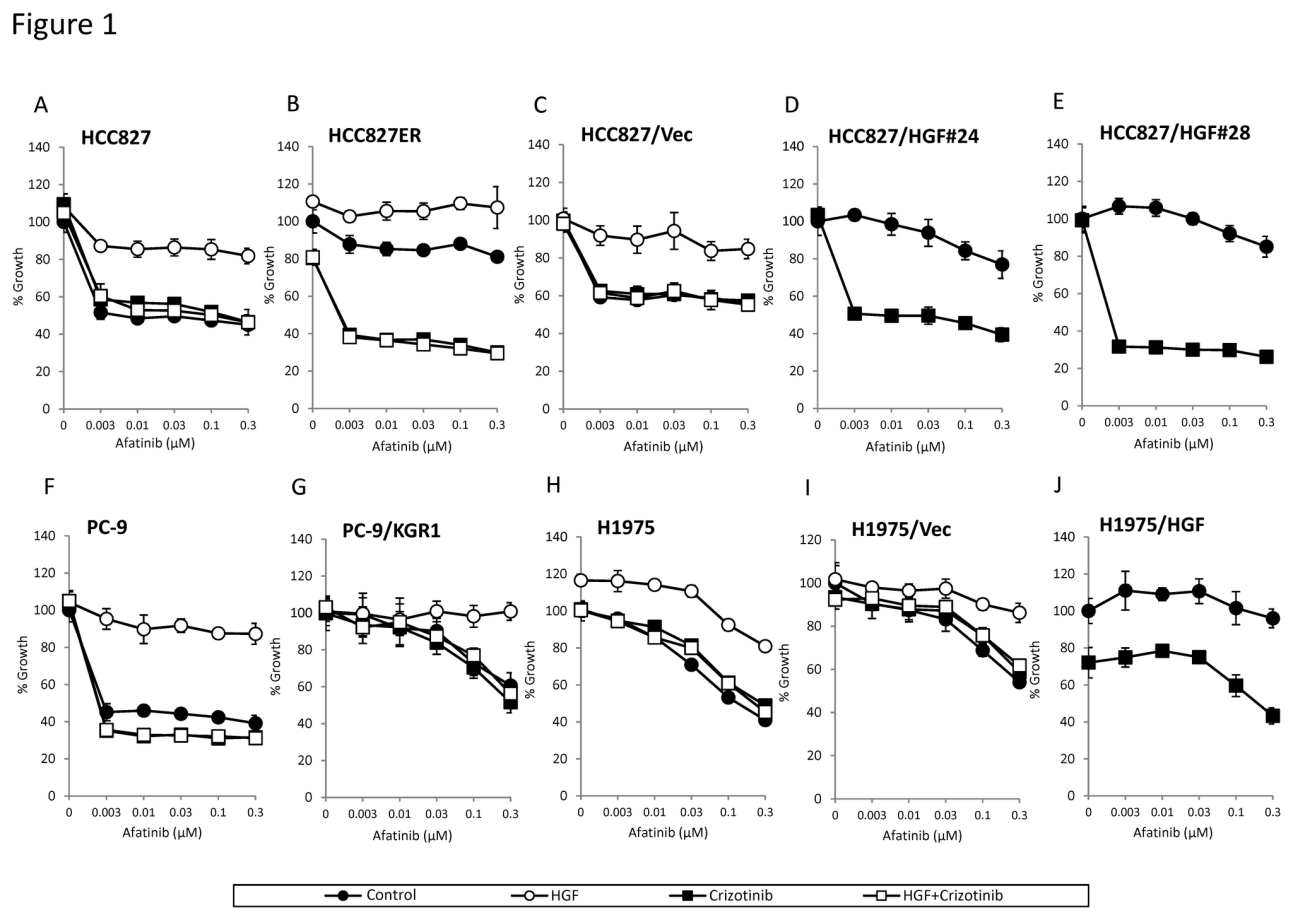
Crizotinib overcomes HGF triggered resistance to irreversible EGFR-TKIs in EGFR-TKI resistant lung cancer cells harboring EGFR mutations. Tumor cells (2×10^3^ cells per well) were incubated with various concentrations of afatinib, with or without crizotinib (300 nmol/L) and/or HGF (10 ng/mL), for 72 hours. Cell growth was determined by the MTT assay. The percentage of growth is shown relative to untreated controls. Each sample was assayed in triplicate, with each experiment repeated at least 3 times independently.

**Figure 2:**
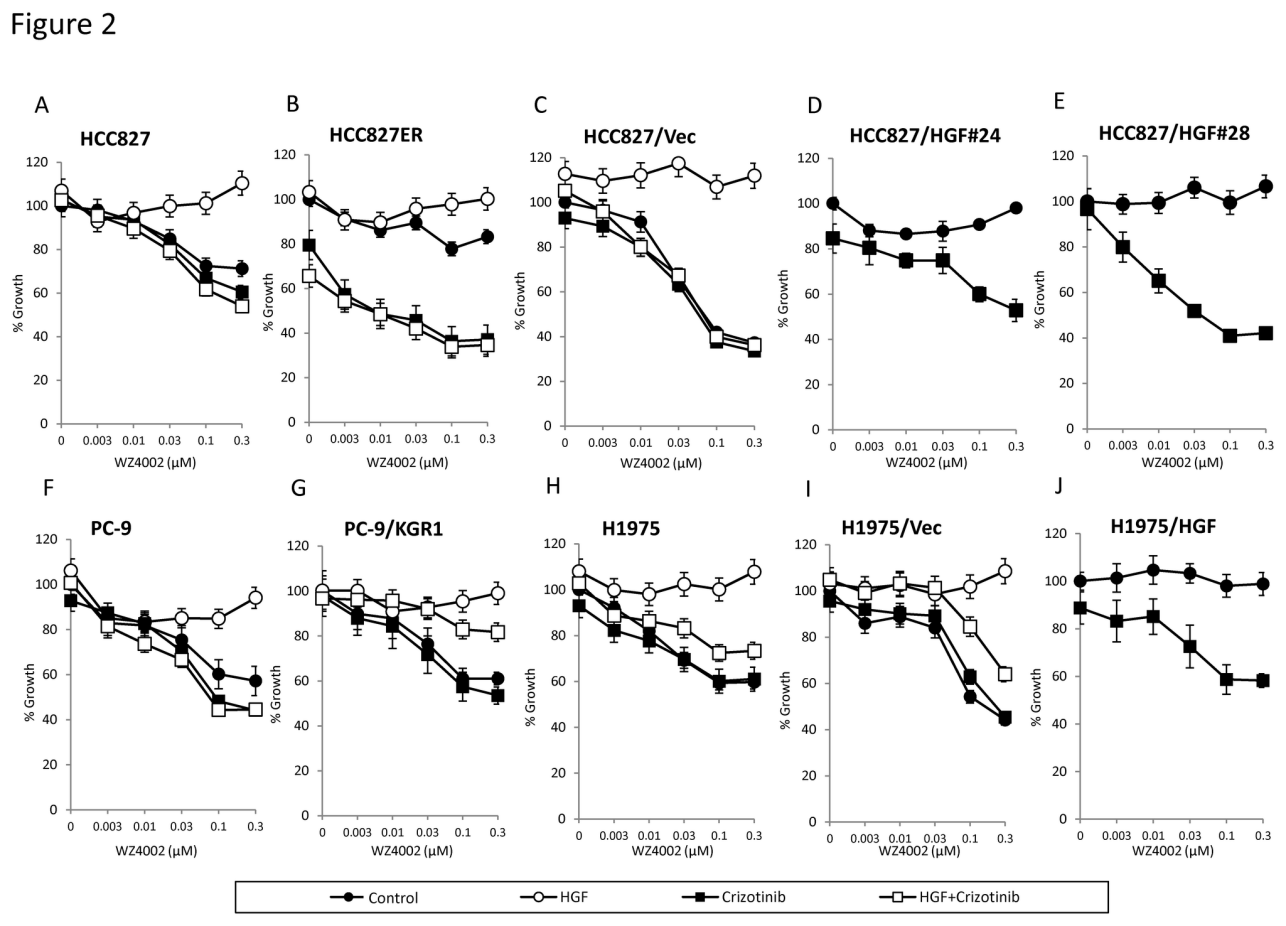
Crizotinib overcomes HGF triggered resistance to mutant-selective EGFR-TKIs in EGFR-TKI resistant lung cancer cells harboring EGFR mutation. Tumor cells (2×10^3^ cells per well) were incubated with various concentrations of WZ4002, with or without crizotinib (300 nmol/L) and/or HGF (10 ng/mL), for 72 hours. Cell growth was determined by the MTT assay. The percentage of growth is shown relative to untreated controls. Each sample was assayed in triplicate, with each experiment repeated at least 3 times independently.

We tested crizotinib plus a new generation EGFR-TKI, irreversible EGFR-TKI afatinib (Figure **1**) or mutant-selective EGFR-TKI WZ4002 (Figure **2**), with HGF induced resistance for several cell lines by the MTT assay. In the first set of experiments, we utilized PC-9 and HCC827 cells, which are human lung adenocarcinoma cell lines with deletions of exon 19 of *EGFR*, resulting in activation of this gene. HCC827 cells were treated with increasing doses of a new generation EGFR-TKI in the presence of HGF alone, crizotinib alone, or HGF pulse crizotinib, and cell proliferation was assessed. While a new generation EGFR-TKI alone reduced cell proliferation, exogenously added HGF caused resistance to a new generation EGFR-TKI treatment in both cell lines. HGF-induced resistance to a new generation EGFR-TKI was abrogated by co-treatment with crizotinib (Figures **1A**, **2A**). Similar results were obtained for PC-9 cells (Figures **1F**, **2F**). We next tested H1975, a human lung adenocarcinoma cell line with both an exon 20 T790M gatekeeper mutation and an exon 21 L858R mutation in *EGFR*; and PC-9/KGR1, a cell line with both an exon 20 T790M gatekeeper mutation and deletions of *EGFR* exon 19. Although both cell lines were resistant to erlotinib (data not shown), they were sensitive to a new generation EGFR-TKI, and HGF induced hyposensitivity in a dose dependent manner (Figure **S1, S2**). Crizotinib sensitized H1975 cells to a new generation EGFR-TKI even in the presence of HGF (Figures **1H**, **2H**). Similar results were obtained for PC-9/KGR1 cells (Figures **1G**, **2G**). We also examined the effect of these compounds in the HCC827ER cell line, with a deletion of *EGFR* exon 19 and *Met* gene amplification. HCC827ER cells were resistant to a new generation EGFR-TKI, and HGF induced mild hyposensitivity. However, crizotinib plus a new generation EGFR-TKI markedly inhibited the growth of HCC827ER cells (Figures **1B**, **2B**). Taken together, these results suggest that crizotinib plus either afatinib or WZ4002 may overcome the resistance to reversible EGFR-TKIs of EGFR mutant lung cancer cells containing an *EGFR* gatekeeper mutation, *Met* gene amplification, and/or HGF overexpression.

We previously reported that, in NSCLC patients, HGF is present mainly in cancer cells with acquired resistance to EGFR-TKIs, suggesting that HGF may be produced predominantly in resistant tumor cells via an autocrine mechanism [25]. To further explore the effect of dual inhibition of EGFR and Met on autocrine production of HGF, we generated stable HGF-gene transfectants in HCC827 (HCC827/HGF#24, and HCC827/HGF#28) and H1975 (H1975/HGF) cells; as a control, we generated HCC827/Vec cells and H1975/Vec cells, which were transfected with vector alone (Table **1**). HCC827/HGF#24, HCC827/HGF#28, and H1975/HGF cells secreted high levels of HGF (284±29, 121±21, and 370±24 ng/ 10^5^ cells/ 24 h, respectively), whereas the concentrations of HGF secreted by HCC827/Vec and H1975/Vec cells were under the limit of detection. HCC827/Vec (Figures **1C**, **2C**) and H1975/Vec (Figures **1I**, **2I**) cells were sensitive to a new generation EGFR-TKI, whereas HCC827/HGF#24 (Figures **1D**, **2D**), HCC827/HGF#28 (Figures **1E**, **2E**), and H1975/HGF (Figures **1J**, **2J**) cells became resistant to these agents. The combination of crizotinib plus afatinib or WZ4002 inhibited the growth of HCC827/HGF#24, HCC827/HGF#28, and H1975/HGF cells. To further confirm that crizotinib acted on Met in cells with HGF-induced resistance to the new generation EGFR-TKIs, we knocked down Met or ALK by specific siRNAs in H1975 cells (Figure **S3**). H1975 cell line expressed no detectable level of wild type ALK or rearranged ALK protein. Treatment with Met, but not ALK, siRNA reversed the HGF-induced resistance to afatinib and WZ4002, indicating that crizotinib acts via the Met pathway in HGF-induced resistance to the new generation EGFR-TKIs. Taken together, these findings indicated that the Met inhibitor crizotinib plus a new generation EGFR-TKI, either afatinib or WZ4002, circumvented resistance to HGF in an autocrine manner, in the presence or absence of the *EGFR* T790M mutation or *Met* gene amplification. 

### Crizotinib overcomes resistance to new generation EGFR-TKIs induced by fibroblast-derived HGF

Because host microenvironments can have a profound effect on the chemosensitivity of cancers and because stromal fibroblasts are the major sources of HGF [32], we assayed HGF production by human fibroblast cell lines. We found that the human embryonic lung-derived fibroblast cell line MRC-5 secreted high levels of HGF into the supernatant. In contrast, HCC827 and H1975 cells did not secrete detectable levels of HGF into the culture supernatant (Table **1**). To further investigate whether the resistance of HCC827 and H1975 cells to afatinib or WZ4002 was affected by crosstalk with stromal fibroblasts, we cocultured lung cancer and MRC-5 cells in Transwell systems. We found that coculture of HCC827 and H1975 cells with MRC-5 cells did not significantly affect the proliferation of the former. In the presence of MRC-5 cells, however, HCC827 and H1975 cells became resistant to afatinib and WZ4002, a resistance abrogated by treatment with crizotinib (100 nmol/L) (Figure **S4**). These results indicate that crizotinib can overcome the resistance to new generation EGFR-TKIs induced by fibroblast-derived HGF in a paracrine manner.

### Crizotinib reduces Met phosphorylation and combined treatment with a new generation EGFR-TKI inhibits downstream pathways even in the presence of HGF

**Figure 3:**
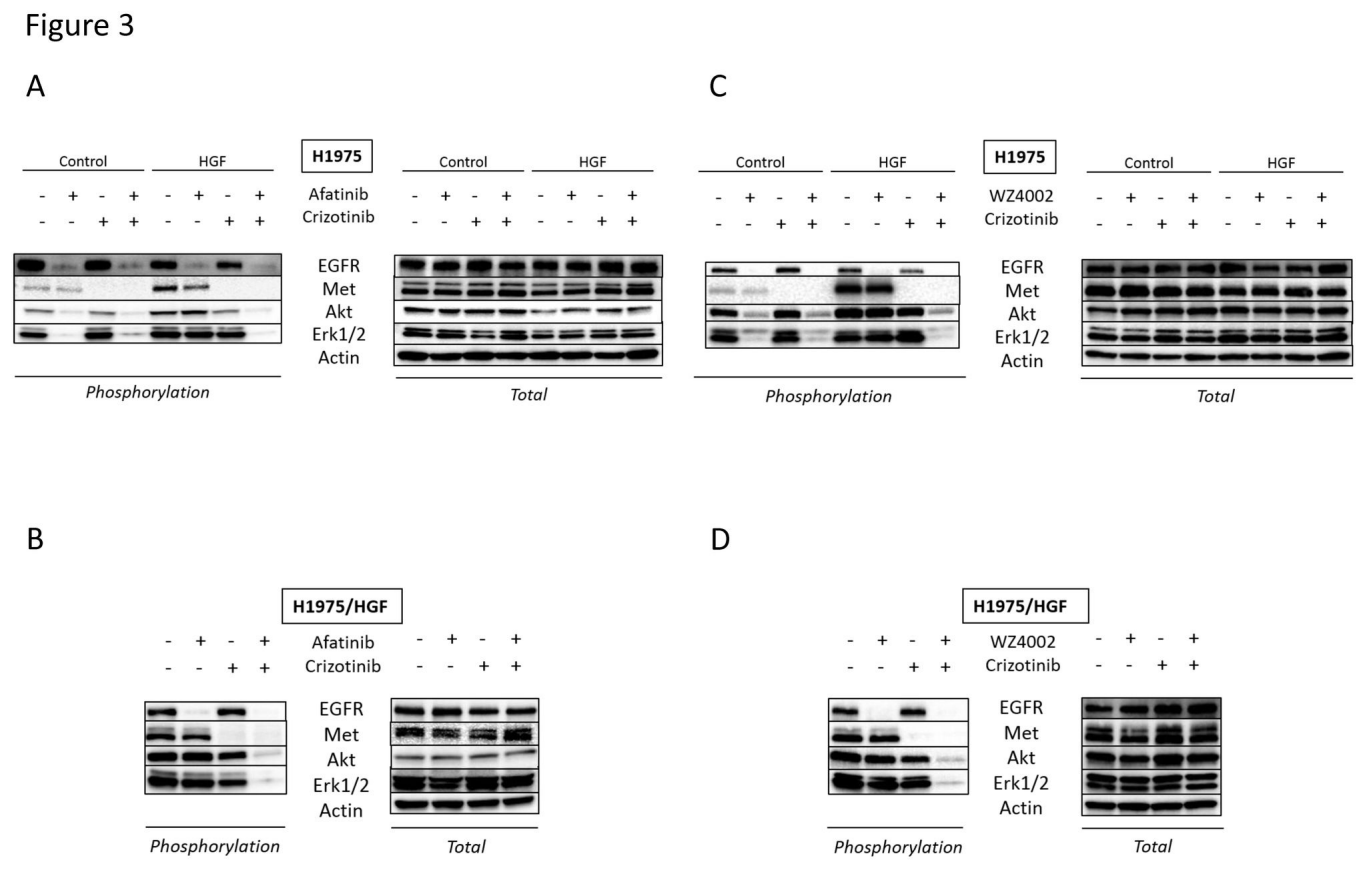
Crizotinib reduces Met phosphorylation and combined treatment with a new generation EGFR-TKI inhibits downstream pathways even in the presence of HGF. H1975 and H1975/HGF cells were incubated with crizotinib (300 nmol/L) and/or afatinib (300 nmol/L) (A, B) or WZ4002 (300 nmol/L) (C, D), for 1 hour. After stimulation with HGF (10 ng/mL) for 10 minutes, the cell lysates were harvested and the phosphorylation of indicated proteins was determined by western blot analysis. Each sample was assayed in triplicate, with each experiment repeated at least 3 times independently.

To explore the molecular mechanism by which combined treatment with crizotinib and afatinib (Figure **3A, 3B**) or WZ4002 (Figure **3B, 3D**) inhibited cell growth in the presence of HGF, we examined the phosphorylation status of Met, EGFR, and their downstream molecules (PI3K/Akt and ERK1/2) in H1975 and H1975/HGF cells by western blotting. These two cells expressed EGFR and Met proteins, both of which were phosphorylated, and the downstream molecules Akt and ERK1/2. While HGF alone did not affect the phosphorylation of EGFR, it stimulated the phosphorylation of Met, thereby activating Akt and ERK1/2. In the absence of HGF, EGFR-TKI inhibited the phosphorylation of EGFR, but not of Met, thereby inhibiting the phosphorylation of Akt and ERK1/2. In the presence of HGF, EGFR-TKI failed to inhibit the phosphorylation of Met, Akt and ERK1/2, although it inhibited EGFR phosphorylation. Thus, the combination of crizotinib and a new generation EGFR-TKI inhibited the phosphorylation of EGFR and Met, and of their receptors and downstream molecules, Akt and ERK1/2, irrespective of the presence of HGF. Similar results were observed in H1975/HGF cells. These results suggested that crizotinib plus the new generation EGFR-TKI overcome resistance to HGF, predominantly by inhibiting the phosphorylation of EGFR and Met proteins, followed by inhibition of downstream Akt and ERK1/2. 

### Combined treatment with crizotinib and a new generation EGFR-TKI overcomes multiple resistance mechanisms to EGFR-TKI *in vivo*

**Figure 4:**
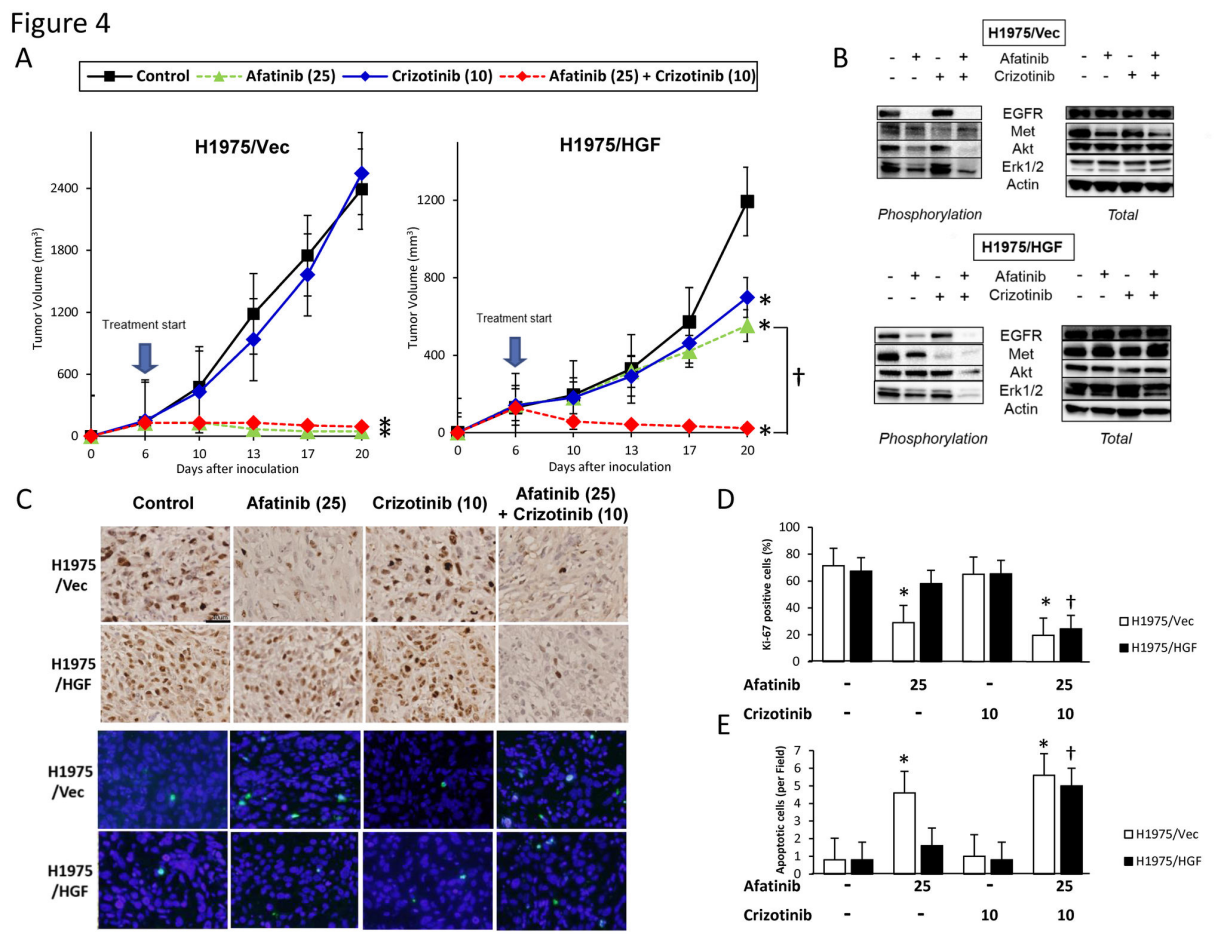
Crizotinib combined with irreversible EGFR-TKI overcomes multiple resistances to EGFR-TKI *in vivo*. (A) SCID mice-bearing H1975/Vec- or H1975/HGF- tumors were administered afatinib (25 mg/kg) and/or crizotinib (10mg/kg) once daily for 6 to 20 days. Tumor volume was measured using calipers on the indicated days. Mean ± SE tumor volumes are shown for groups of 5 mice. *, P < 0.05 versus control; ✝, P < 0.05 versus afatinib (25 mg/kg) by one-way ANOVA. (B) H1975/Vec- or H1975/HGF- tumors were resected from the mice 3 hours after administration of afatinib (25mg/kg) and/or crizotinib (10 mg/kg), and the relative levels of proteins in the tumor lysates were determined by western blot analysis. (C) Representative images of H1975/Vec- and H1975/HGF tumors immunohistochemically stained with antibodies to human Ki-67, and stained with both DAPI (nuclear stain) and TUNEL (FITC). Bar, 200 μm. (D) Quantification of proliferative cells, as determined by their Ki-67-positive proliferation index (percentage of Ki-67-positive cells). Quantification of apoptotic cells, as determined by the TUNEL assay as described in Materials and Methods. Columns, mean of five areas; bars, SD. *, P < 0.05 versus H1975/Vec-tumors; ✝, P < 0.05 versus control of H1975/HGF-tumors by one-way ANOVA.

**Figure 5:**
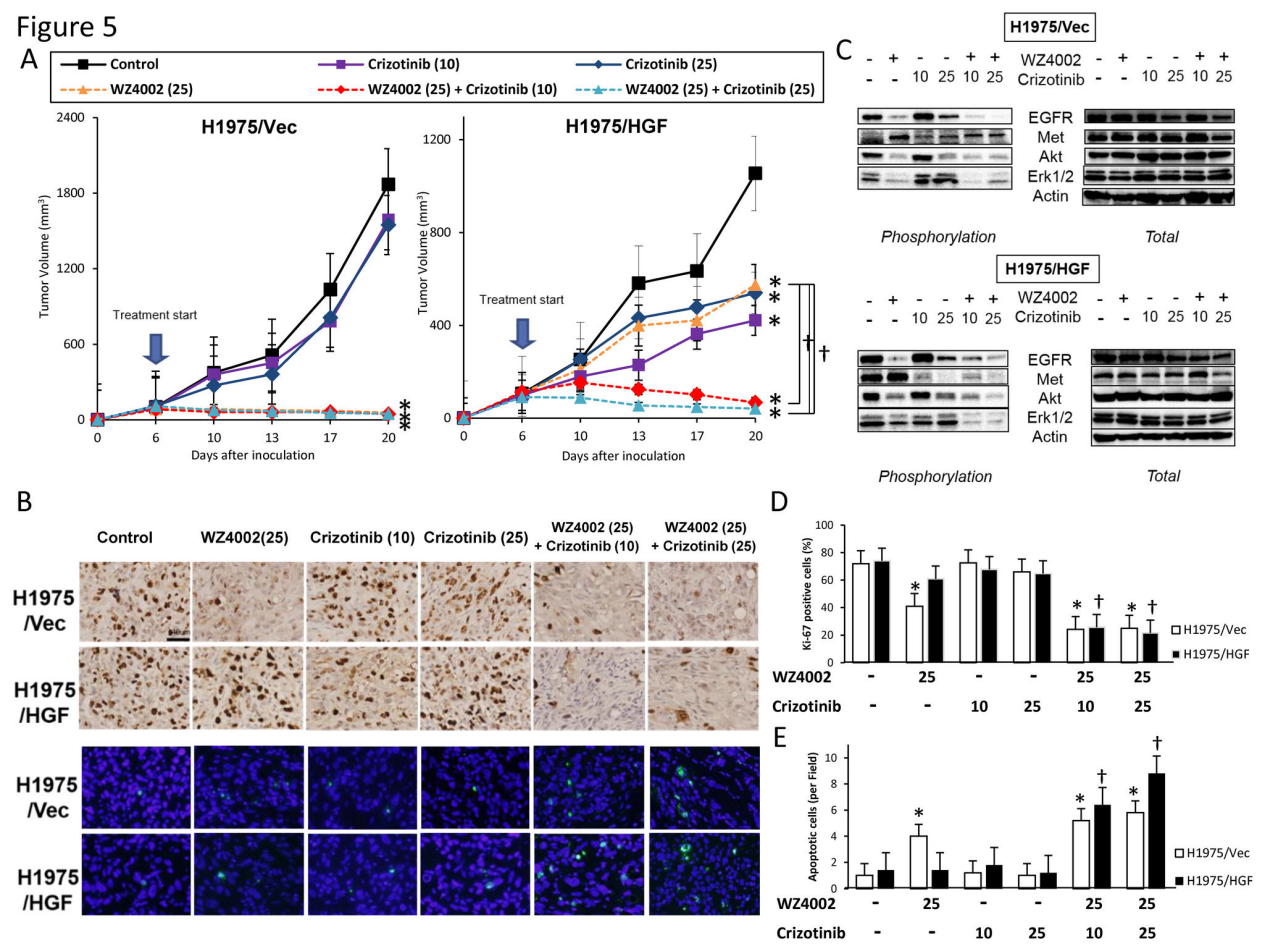
Crizotinib combined with mutant-selective EGFR-TKI overcomes multiple resistances to EGFR-TKI *in* *vivo*. (A) SCID mice-bearing H1975/Vec- or H1975/HGF- tumors were administered WZ4002 (25 mg/kg) and/or crizotinib (10, 25mg/kg) once daily for 6 to 20 days. Tumor volume was measured using calipers on the indicated days. Mean ± SE tumor volumes are shown for groups of 5 mice. *, P < 0.05 versus control; ✝, P < 0.05 versus WZ4002 by one-way ANOVA. (B) H1975/Vec- or H1975/HGF- tumors were resected from the mice 3 hours after administration of WZ4002 (25mg/kg) and/or crizotinib (10, 25 mg/kg), and the relative levels of proteins in the tumor lysates were determined by western blot analysis. (C) Representative images of H1975/Vec- and H1975/HGF- tumors immunohistochemically stained with antibodies to human Ki-67, and stained with both DAPI (nuclear stain) and TUNEL (FITC). Bar, 200 μm. (D) Quantification of proliferative cells, as determined by the Ki-67-positive proliferation index (percentage of Ki-67-positive cells). Quantification of apoptotic cells, as determined by the TUNEL assay as described in Materials and Methods. Columns, mean of five areas; bars, SD *, P < 0.05 versus of H1975/Vec-tumors; ✝, P < 0.05 versus H1975/HGF-tumors by one-way ANOVA.

We next evaluated whether crizotinib plus afatinib (Figure **4A**) or WZ4002 (Figure **5A**) could overcome resistance to EGFR-TKI caused by the gatekeeper *EGFR*-T790M mutation and/or HGF overexpression *in vivo*. Oral administration to mice of afatinib or WZ4002, with or without crizotinib, but not crizotinib alone, markedly inhibited the growth of H1975/Vec-tumors with the gatekeeper *EGFR*-T790M mutation. None of these three agents alone inhibited the growth of H1975/HGF-tumors, which both overexpress HGF and have the gatekeeper *EGFR*-T790M mutation, indicating that HGF induced resistance to the new generation EGFR-TKIs *in vivo*. In contrast, the combination of afatinib or WZ4002 plus 10mg/kg crizotinib markedly suppressed the growth of H1975/HGF tumors. A greater degree of growth suppression was observed with WZ4002 plus 25mg/kg crizotinib than WZ4002 plus 10mg/kg crizotinib (Figure **5A**). These results indicated that the addition of crizotinib may overcome *in vivo* resistance to the new generation of EGFR-TKIs induced by HGF overexpression and the *EGFR*-T790M mutation.

### Combined treatment with crizotinib and a new generation EGFR-TKI decreases cell proliferation and increases cell apoptosis *in vivo* by inhibiting of EGFR and Met phosphorylation

To assess the mechanism of action of combined therapy, we assayed the phosphorylation status of target molecules in H1975/Vec- and H1975/HGF-induced tumors by western blotting (Figure **4B**, **5B**). EGFR-TKIs inhibited the phosphorylation of EGFR, Akt, and ERK1/2 in H1975/Vec-, but not in H1975/HGF-induced tumors. The combination of afatinib (Figure **4B**) or WZ4002 (Figure **5B**) plus crizotinib inhibited the phosphorylation of EGFR and Met proteins, as well as the phosphorylation of their receptors and downstream molecules, Akt and ERK1/2, in mouse tumors induced by H1975/Vec and H1975/HGF cells. These results suggested that crizotinib plus a new generation EGFR-TKI can overcome resistance to a new generation EGFR-TKI by inhibiting the phosphorylation of EGFR and Met proteins, followed by inhibiting downstream Akt and ERK1/2 *in vivo*.

To further assess the mechanism of action of combined therapy, we assayed proliferation (Figure **4C, 4D**, **5C, 5D**) and apoptosis (Figure **4C, 4E**, **5C, 5E**) in H1975/Vec- and H1975/HGF-induced tumors. Treatment of mice carrying H1975/HGF-tumors with afatinib (Figure **4C, 4D, 4E**) or WZ4002 (Figure **5C, 5D, 5E**), but not with crizotinib alone, significantly reduced the number of proliferating cells and increased the number of apoptotic cells compared with control, untreated mice. Similarly, treatment with crizotinib plus afatinib (Figure **4A**) or WZ4002 (Figure **5A**) had a greater effect than any agent alone, with the greatest effect observed with WZ4002 plus high dose crizotinib. These results suggested that the combination use of a new generation EGFR-TKI and crizotinib inhibited tumor growth by decreasing cell proliferation and increasing cell apoptosis. 

### Toxicity profiles of new generation EGFR-TKIs plus crizotinib

**Figure 6:**
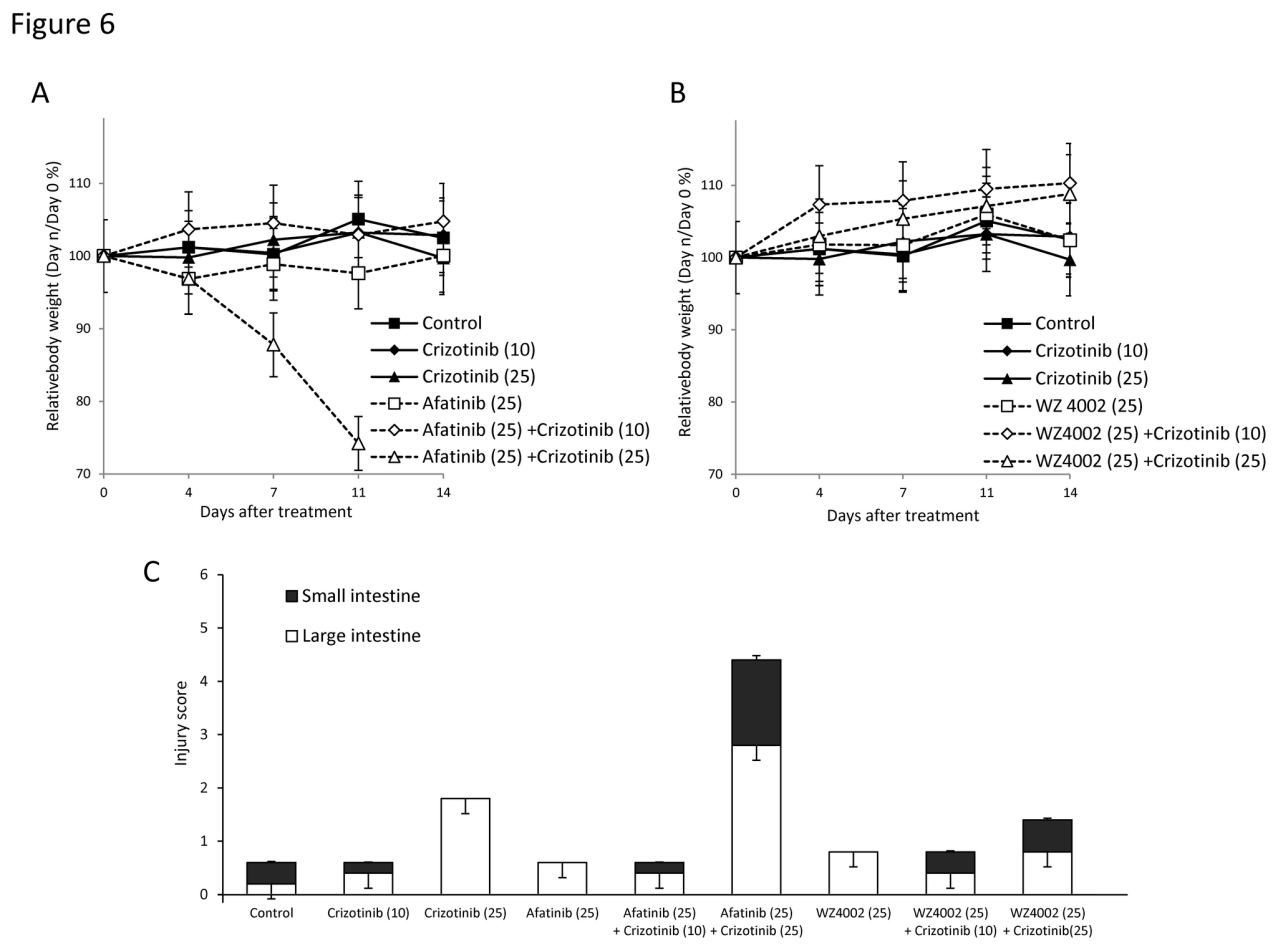
Toxicity profiles of crizotinib plus afatinib or WZ4002 *in* *vivo*. (A), (B), Relative body weight of mice during treatment with afatinib or WZ4002 and/or crizotinib. Mean ± SD body weights are shown for groups of 5 mice. (C), Quantification of mucosal damage by injury index of intestinal sections (black column: large intestine, white column: small intestine), as determined by H&E staining. Each column represents the mean of two areas for groups of 5 mice; bars, SD.

To evaluate the toxicities of afatinib or WZ4002 when combined with crizotinib, we analyzed mouse body weight (Figure **6A, 6B**) and intestinal mucosal damage (Figure **6C**, Figure **S5, S6**) during treatment *in vivo*. Histological analysis showed that the combination of afatinib and high dose crizotinib (25mg/kg/day) induced lethal toxicity, including severe intestinal mucosal damage and loss of body weight (Figure **6A, 6C**), indicating that long-term treatment with the combination of an irreversible EGFR-TKI and crizotinib may cause severe, even fatal, toxicities. In contrast, the combination of the mutant-selective EGFR-TKI WZ4002 and high or low dose of crizotinib had little effect on intestinal mucosal histology or mouse weight (Figure **6B, 6C**). Treatment with low dose crizotinib alone, afatinib alone, or WZ4002 alone had little effect on the intestinal mucosa and mouse weight, suggesting that combination therapy, not any agent alone, causes severe toxicities. 

## Discussion

Many attempts have been made to overcome the resistance of lung cancers refractory to reversible EGFR-TKIs and harboring EGFR activating mutations. Although irreversible EGFR-TKIs such as afatinib have been tested in clinical trials for EGFR-TKI-refractory lung cancer, monotherapy with agents of this class has shown minimum benefits with severe adverse effects [32]. Of patients with *EGFR* mutant lung cancer, 26-40% had tumors with high HGF expression and *EGFR*-T790M secondary mutation, 5-33% had tumors with *Met* gene amplification and *EGFR*-T790M secondary mutation, and 4-7% had tumors with high HGF expression and *Met* gene amplification, suggesting that dual targeting of HGF/Met and the *EGFR*-T790M mutation may overcome resistance to EGFR-TKIs [23,25,33]. 

HGF was originally identified as a hepatocyte mitogen and has since been shown to have pleiotropic biological activities [29]. HGF and its receptor Met are expressed at various levels in various types of cancer cells [24]. Many lung cancer cells express Met, with these cells and others in their microenvironment expressing their Met ligands [34], suggesting that these receptors and ligands modulate the sensitivity of cancer cells to molecular targeted drugs in their microenvironment. 

The lack of response of EGFR-TKI resistance tumors to monotherapy may be caused by the heterogeneity of resistance mechanisms. We therefore assessed methods to overcome resistance to multiple drugs caused by EGFR and/or Met signaling without causing severe adverse effects. Here, we focused on crizotinib as a Met inhibitor. Although approved by the U.S. Food and Drug Administration as an ALK inhibitor, crizotinib was found to be a potent Met inhibitor, with an IC50 for wild type c-Met of 4 nM. Moreover, this agent was clinically safe, suggesting that it may be a candidate for overcoming the HGF-Met axis induced resistance to reversible EGFR-TKIs. 

Dual blockade of HGF/Met and mutant EGFR was shown to overcome the resistance to EGFR-TKIs caused by *EGFR*-T790M mutation and *Met* gene amplification in a preclinical model [33]. We have extended these findings, showing that crizotinib plus afatinib or WZ4002 could overcome EGFR-TKI caused by HGF overexpression in both autocrine and paracrine systems, as well as resistances caused by the gatekeeper *EGFR*-T790M mutation and *Met* gene amplification. Dual blockade of HGF/Met and mutant EGFR may therefore overcome concurrent resistance to EGFR-TKIs. The strongest anti-tumor effects were exhibited by the combination of high dose crizotinib and a new generation EGFR-TKI, which reduced tumor proliferation and increased tumor apoptosis *in vivo*, indicating that complete dual blockade of mutant EGFR and Met may overcome resistance to EGFR-TKIs.

Importantly, we observed severe toxicity, such as intestinal mucosal damage and weight loss, when high dose crizotinib and afatinib were combined. These lethal toxicities were not observed when WZ4002 was combined with crizotinib, suggesting that afatinib, but not WZ4002, inhibited wild type EGFR which express in the intestinal mucosa. Moreover, these findings indicated that adverse effects should be carefully evaluated in clinical trials with combinations of agents targeting both EGFR and Met.

Several strategies have been proposed to overcome resistance to reversible EGFR-TKIs, including treatment with afatinib, an anti-EGFR antibody [35], Hsp90 inhibitors [36], PI3K/mTOR inhibitor [37], and mutant-selective EGFR-TKIs [22]. Of them, mutant-selective EGFR-TKIs have shown activity not only against tumors harboring exon19 deletions and the L858R mutation, but against tumors with the T790M resistance mutation. In addition, these agents may be less toxic than traditional EGFR-TKIs since they target EGFR carrying only certain specific mutations. Further clinical development of this class of inhibitors in EGFR-mutant lung cancer patients who become refractory to reversible EGFR-TKIs is warranted. 

In summary, we found that crizotinib combined with a new generation EGFR-TKI may overcome multiple resistances of lung tumors to reversible EGFR-TKIs. These agents may inhibit tumor proliferation and promote tumor apoptosis via blockade of both mutant EGFR and Met signaling. These findings suggest that treatment with crizotinib plus a new generation EGFR-TKI, especially one selective for mutant EGFR, may result in more successful outcomes in lung cancers with resistance to EGFR-TKIs through the mutant EGFR and/or HGF/Met pathways.

## Supporting Information

Figure S1 **HGF dose-dependently induced resistance to afatinib in lung cancer cells harboring EGFR mutations.** PC-9, HCC827, PC-9/KGR1, and H1975 cells (2×10^3^ cells per well) were incubated with various concentrations of afatinib and HGF (0, 10, 20, 50 ng/mL) for 72 hours. Cell growth was determined by the MTT assay. The percentage of growth is shown relative to untreated controls. Each sample was assayed in triplicate, with each experiment repeated at least 3 times independently.
(TIF)

Figure S2 **HGF dose-dependently induced resistance to WZ4002 in lung cancer cells harboring EGFR mutations.** PC-9, HCC827, PC-9/KGR1, and H1975 cells (2×10^3^ cells per well) were incubated with various concentrations of WZ4002 and HGF (0, 10, 20, 50 ng/mL) for 72 hours. Cell growth was determined by the MTT assay. The percentage of growth is shown relative to untreated controls. Each sample was assayed in triplicate, with each experiment repeated at least 3 times independently.
(TIF)

Figure S3 **Specific downregulation of Met, but not ALK, reversed afatinib (300nmol/L) or WZ4002 (300nmol/L) resistance induced by HGF (10ng/mL) in H1975 cells.** The percentage of growth is shown relative to untreated controls. Each sample was assayed in triplicate, with each experiment repeated at least 3 times independently. *, P < 0.05 by one-way ANOVA. Downregulation of Met or ALK by specific-siRNA was assessed by immunoblotting.
(TIF)

Figure S4 **Crizotinib overcomes resistance to new generation EGFR-TKIs caused by fibroblast-derived HGF.** Tumor cells (8 × 10^3^ cells/800 μL) were cultured with or without afatinib (100 nmol/L) (A) or WZ4002 (100nmol/L) (B) in the lower chambers of Transwell Collagen-Coated chambers. MRC-5 cells (1 × 10^4^ cells/300 μL), which were or were not pretreated for 2 hours with anti-human HGF antibody (5 μg/mL) or crizotinib (100 nmol/L) were placed in the upper chambers, and the cells were cocultured for 72 hours. The number of cells in the lower chamber was determined by the MTT assay. Percent growth was relative to untreated controls. All samples were assayed at least in triplicate, with each experiment performed three times independently. *, P < 0.05 by one-way ANOVA.
(TIF)

Figure S5 **Representative mucosal damage to the small intestine, as assessed by H&E staining.**
(TIF)

Figure S6 **Representative mucosal damage to the large intestine, as assessed by H&E staining.**
(TIF)

## References

[1] Pao W, Chmielecki J (2010) Rational, biologically based treatment of EGFR-mutant non-small-cell lung cancer. Nat Rev Cancer 10: 760–774. doi:10.1038/nrc2947. PubMed: 20966921. 20966921 PMC3072803

[2] Inoue A, Kobayashi K, Maemondo M, Sugawara S, Oizumi S et al. (2013) Updated overall survival results from a randomized phase III trial comparing gefitinib with carboplatin-paclitaxel for chemo-naïve non-small cell lung cancer with sensitive EGFR gene mutations (NEJ002). Ann Oncol 24: 54-49. doi:10.1093/annonc/mdt459.103. PubMed: 22967997. 22967997

[3] Mitsudomi T, Morita S, Yatabe Y, Negoro S, Okamoto I et al. (2010) Gefitinib versus cisplatin plus docetaxel in patients with non-small-cell lung cancer harbouring mutations of the epidermal growth factor receptor (WJTOG3405): an open label, randomised phase 3 trial. Lancet Oncol 11: 121–128. doi:10.1016/S1470-2045(09)70364-X. PubMed: 20022809. 20022809

[4] Rosell R, Carcereny E, Gervais R, Vergnenegre A, Massuti B et al. (2012) Erlotinib versus standard chemotherapy as first-line treatment for European patients with advanced EGFR mutation-positive non-small-cell lung cancer (EURTAC): a multicentre, open-label, randomised phase 3 trial. Lancet Oncol 13: 239-246. doi:10.1016/S1470-2045(12)70227-9. PubMed: 22285168. 22285168

[5] Sequist LV, Yang JC, Yamamoto N, O'Byrne K, Hirsh V et al. (2013) Phase III Study of Afatinib or Cisplatin Plus Pemetrexed in Patients With Metastatic Lung Adenocarcinoma With EGFR Mutations. J Clin Oncol 31(27):3327-34. PubMed: 23816960. 23816960 10.1200/JCO.2012.44.2806

[6] Mitsudomi T, Yatabe Y (2007) Mutations of the epidermal growth factor receptor gene and related genes as determinants of epidermal growth factor receptor tyrosine kinase inhibitors sensitivity in lung cancer. Cancer Sci 98: 1817–1824. doi:10.1111/j.1349-7006.2007.00607.x. PubMed: 17888036. 17888036 PMC11159145

[7] Kobayashi S, Boggon TJ, Dayaram T, Jänne PA, Kocher O et al. (2005) EGFR mutation and resistance of non-small-cell lung cancer to gefitinib. N Engl J Med 352: 786-792. doi:10.1056/NEJMoa044238. PubMed: 15728811. 15728811

[8] Pao W, Miller VA, Politi KA, Riely GJ, Somwar R et al. (2005) Acquired resistance of lung adenocarcinomas to gefitinib or erlotinib is associated with a second mutation in the EGFR kinase domain. PLoS Med 2: e73. doi:10.1371/journal.pmed.0020073. PubMed: 15737014. 15737014 PMC549606

[9] Engelman JA, Zejnullahu K, Mitsudomi T, Song Y, Hyland C et al. (2007) MET amplification leads to gefitinib resistance in lung cancer by activating ERBB3 signaling. Science 316: 1039-1043. doi:10.1126/science.1141478. PubMed: 17463250. 17463250

[10] Yano S, Wang W, Li Q, Matsumoto K, Sakurama H et al. (2008) Hepatocyte growth factor induces gefitinib resistance of lung adenocarcinoma with epidermal growth factor receptor-activating mutations. Cancer Res 68: 9479-9487. doi:10.1158/0008-5472.CAN-08-1643. PubMed: 19010923. 19010923

[11] Zhang Z, Lee JC, Lin L, Olivas V, Au V et al. (2012) Activation of the AXL kinase causes resistance to EGFR-targeted therapy in lung cancer. Nat Genet 44: 852-860. doi:10.1038/ng.2330. PubMed: 22751098. 22751098 PMC3408577

[12] Ludovini V, Bianconi F, Pistola L, Chiari R, Minotti V et al. (2011) Phosphoinositide-3-kinase catalytic alpha and KRAS mutations are important predictors of resistance to therapy with epidermal growth factor receptor tyrosine kinase inhibitors in patients with advanced non-small cell lung cancer. J Thorac Oncol 6: 707-715. doi:10.1097/JTO.0b013e31820a3a6b. PubMed: 21258250. 21258250

[13] Yamamoto C, Basaki Y, Kawahara A, Nakashima K, Kage M et al. (2010) Loss of PTEN expression by blocking nuclear translocation of EGR1 in gefitinib-resistant lung cancer cells harboring epidermal growth factor receptor-activating mutations. Cancer Res 70: 8715-8725. doi:10.1158/0008-5472.CAN-10-0043. PubMed: 20959484. 20959484

[14] Sequist LV, Waltman BA, Dias-Santagata D, Digumarthy S, Turke AB et al. (2011) Genotypic and histological evolution of lung cancers acquiring resistance to EGFR inhibitors. Sci Transl Med 3: 75ra26 PubMed: 21430269. 10.1126/scitranslmed.3002003 PMC3132801 21430269

[15] Suda K, Tomizawa K, Fujii M, Murakami H, Osada H et al. (2011) Epithelial to mesenchymal transition in an epidermal growth factor receptor-mutant lung cancer cell line with acquired resistance to erlotinib. J Thorac Oncol 6: 1152-1161. doi:10.1097/JTO.0b013e318216ee52. PubMed: 21597390. 21597390

[16] Yun CH, Mengwasser KE, Toms AV, Woo MS, Greulich H et al. (2008) The T790M mutation in EGFR kinase causes drug resistance by increasing the affinity for ATP. Proc Natl Acad Sci U S A 105: 2070-2075. doi:10.1073/pnas.0709662105. PubMed: 18227510. 18227510 PMC2538882

[17] Kwak EL, Sordella R, Bell DW, Godin-Heymann N, Okimoto RA et al. (2005) Irreversible inhibitors of the EGF receptor may circumvent acquired resistance to gefitinib. Proc Natl Acad Sci U S A 102: 7665–7670. doi:10.1073/pnas.0502860102. PubMed: 15897464. 15897464 PMC1129023

[18] Kobayashi S, Ji H, Yuza Y, Meyerson M, Wong KK et al. (2005) An alternative inhibitor overcomes resistance caused by a mutation of the epidermal growth factor receptor. Cancer Res 65: 7096–7101. doi:10.1158/0008-5472.CAN-05-1346. PubMed: 16103058. 16103058

[19] Yu Z, Boggon TJ, Kobayashi S, Jin C, Ma PC et al. (2007) Resistance to an irreversible epidermal growth factor receptor (EGFR) inhibitor in EGFR-mutant lung cancer reveals novel treatment strategies. Cancer Res 67: 10417–10427. doi:10.1158/0008-5472.CAN-07-1248. PubMed: 17974985. 17974985

[20] Engelman JA, Zejnullahu K, Gale CM, Lifshits E, Gonzales AJ et al. (2007) PF00299804, an irreversible pan-ERBB inhibitor, is effective in lung cancer models with EGFR and ERBB2 mutations that are resistant to gefitinib. Cancer Res 67: 11924–11932. doi:10.1158/0008-5472.CAN-07-1885. PubMed: 18089823. 18089823

[21] Li D, Ambrogio L, Shimamura T, Kubo S, Takahashi M et al. (2008) BIBW2992, an irreversible EGFR/HER2 inhibitor highly effective in preclinical lung cancer models. Oncogene 27: 4702–4711. doi:10.1038/onc.2008.109. PubMed: 18408761. 18408761 PMC2748240

[22] Zhou W, Ercan D, Chen L, Yun CH, Li D et al. (2009) Novel mutant-selective EGFR kinase inhibitors against EGFR T790M. Nature 462: 1070-1074. doi:10.1038/nature08622. PubMed: 20033049. 20033049 PMC2879581

[23] Turke AB, Zejnullahu K, Wu YL, Song Y, Dias-Santagata D et al. (2010) Preexistence and clonal selection of MET amplification in EGFR mutant NSCLC. Cancer Cell 17: 77-88. doi:10.1016/j.ccr.2009.11.022. PubMed: 20129249. 20129249 PMC2980857

[24] Yamada T, Matsumoto K, Wang W, Li Q, Nishioka Y et al. (2010) Hepatocyte growth factor reduces susceptibility to an irreversible epidermal growth factor receptor inhibitor in EGFR-T790M mutant lung cancer. Clin Cancer Res 16: 174-183. doi:10.1158/1078-0432.CCR-09-1204. PubMed: 20008840. 20008840

[25] Yano S, Yamada T, Takeuchi S, Tachibana K, Minami Y et al. (2011) Hepatocyte growth factor expression in EGFR mutant lung cancer with intrinsic and acquired resistance to tyrosine kinase inhibitors in a Japanese cohort. J Thorac Oncol 6: 2011-2017. doi:10.1097/JTO.0b013e31823ab0dd. PubMed: 22052230. 22052230

[26] Kwak EL, Bang YJ, Camidge DR, Shaw AT, Solomon B et al. (2010) Anaplastic lymphoma kinase inhibition in non-small-cell lung cancer. N Engl J Med 363: 1693–1703. doi:10.1056/NEJMoa1006448. PubMed: 20979469. 20979469 PMC3014291

[27] Suda K, Murakami I, Katayama T, Tomizawa K, Osada H et al. (2010) Reciprocal and complementary role of MET amplification and EGFR T790M mutation in acquired resistance to kinase inhibitors in lung cancer. Clin Cancer Res 16: 5489–5498. doi:10.1158/1078-0432.CCR-10-1371. PubMed: 21062933. 21062933

[28] Yun CH, Mengwasser KE, Toms AV, Woo MS, Greulich H et al. (2008) The T790M mutation in EGFR kinase causes drug resistance by increasing the affinity for ATP. Proc Natl Acad Sci U S A 105: 2070–2075. doi:10.1073/pnas.0709662105. PubMed: 18227510. 18227510 PMC2538882

[29] Nakamura T, Nishizawa T, Hagiya M, Seki T, Shimonishi M et al. (1989) Molecular cloning and expression of human hepatocyte growth factor. Nature 342: 440–443. doi:10.1038/342440a0. PubMed: 2531289. 2531289

[30] Nishioka Y, Yano S, Fujiki F, Mukaida N, Matsushima K et al. (1997) Combined therapy of multidrug-resistant human lung cancer with anti-P-glycoprotein antibody and monocyte chemoattractant protein-1 gene transduction: the possibility of immunological overcoming of multidrug resistance. Int J Cancer 71: 170-177. doi:10.1002/(SICI)1097-0215(19970410)71:2. PubMed: 9139838. 9139838

[31] ; Green LM, Reade JL, Ware CF (1984) Rapid colorimetric assay for cell viability: application to the quantitation of cytotoxic and growth inhibitory lymphokines. J Immunol Methods 70: 257–268. doi:10.1016/0022-1759(84)90190-X. PubMed: 6609997. Available online at: 10.1016/0022-1759(84)90190-X Available online at: PubMed: 6609997 6609997

[32] Miller VA, Hirsh V, Cadranel J, Chen YM, Park K et al. (2012) Afatinib versus placebo for patients with advanced, metastatic non-small-cell lung cancer after failure of erlotinib, gefitinib, or both, and one or two lines of chemotherapy (LUX-Lung 1): a phase 2b/3 randomised trial. Lancet Oncol 13: 528-538. doi:10.1016/S1470-2045(12)70087-6. PubMed: 22452896. 22452896

[33] Xu L, Kikuchi E, Xu C, Ebi H, Ercan D et al. (2012) Combined EGFR/MET or EGFR/HSP90 inhibition is effective in the treatment of lung cancers codriven by mutant EGFR containing T790M and MET. Cancer Res 72: 3302-3311. doi:10.1158/1538-7445.AM2012-3302. PubMed: 22552292. 22552292 PMC3389159

[34] Matsumoto K, Nakamura T (2006) Hepatocyte growth factor and the Met system as a mediator of tumor-stromal interactions. Int J Cancer 119: 477- 483. doi:10.1002/ijc.21808. PubMed: 16453287. 16453287

[35] ; Regales L, Gong Y, Shen R, de Stanchina E, Vivanco I et al. (2009) Dual targeting of EGFR can overcome a major drug resistance mutation in mouse models of EGFR mutant lung cancer. J Clin Invest 119: 3000–3010. PubMed: 19759520. Available online at: PubMed: 19759520 19759520 10.1172/JCI38746 PMC2752070

[36] Shimamura T, Li D, Ji H, Haringsma HJ, Liniker E et al. (2008) Hsp90 inhibition suppresses mutant EGFR-T790M signaling and overcomes kinase inhibitor resistance. Cancer Res 68: 5827–5838. doi:10.1158/0008-5472.CAN-07-5428. PubMed: 18632637. 18632637 PMC3272303

[37] Sano T, Takeuchi S, Nakagawa T, Ishikawa D, Nanjo S et al. (2013) The novel phosphoinositide 3-kinase–mammalian target of rapamycin inhibitor, BEZ235, circumvents erlotinib resistance of epidermal growth factor receptor mutant lung cancer cells triggered by hepatocyte growth factor. Int J Cancer 133: 505–514. doi:10.1002/ijc.28034. PubMed: 23319394. 23319394

