# Supplementary figures and images for: Ability of the Met Kinase Inhibitor Crizotinib and New Generation EGFR Inhibitors to Overcome Resistance to EGFR Inhibitors
Source: PLoS One. 2013 Dec 26;8(12):e84700. doi: 10.1371/journal.pone.0084700 (PMC3873434; doi:10.1371/journal.pone.0084700)

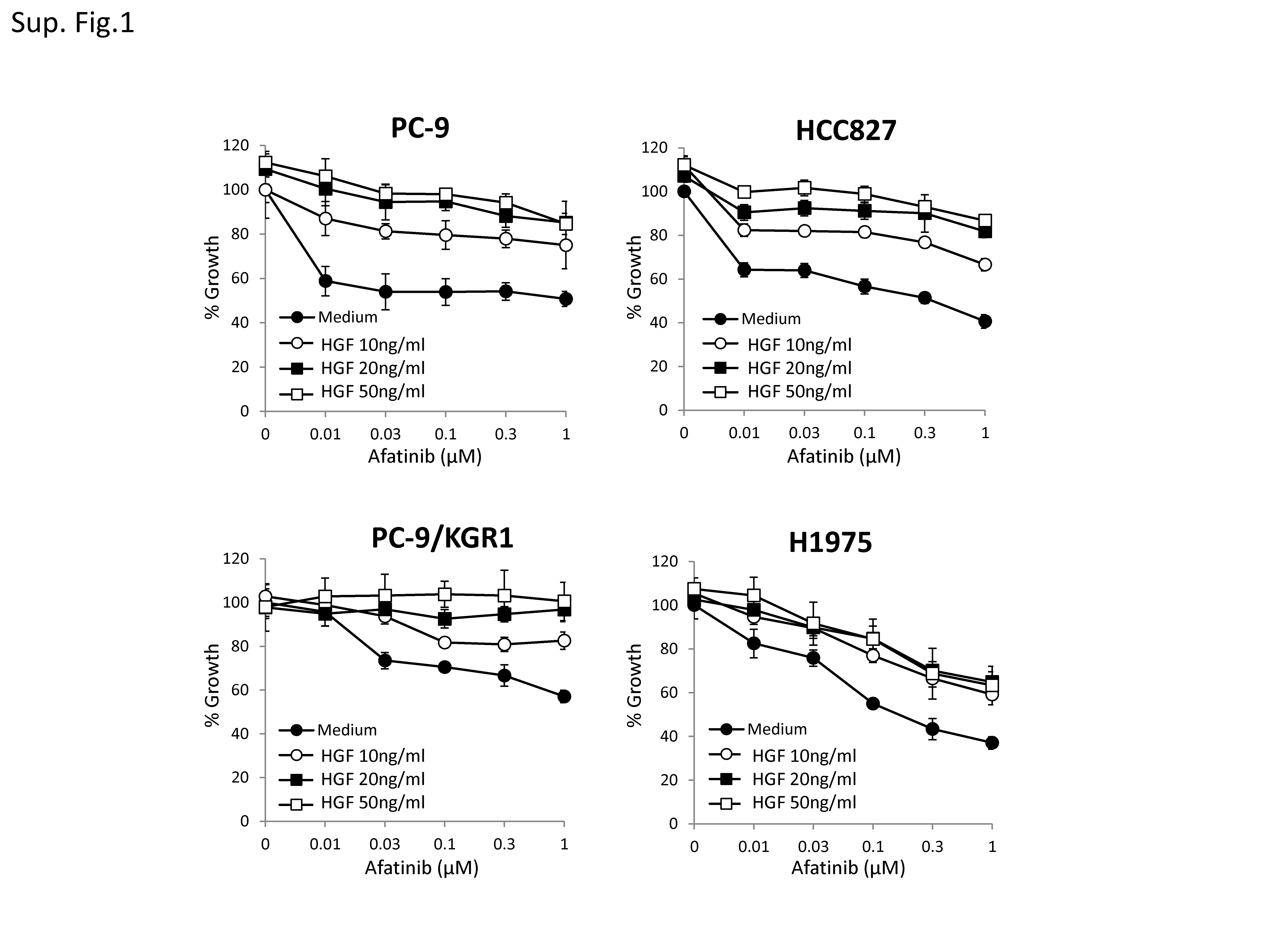

Supplement: Figure S1 — HGF dose-dependently induced resistance to afatinib in lung cancer cells harboring EGFR mutations. PC-9, HCC827, PC-9/KGR1, and H1975 cells (2×10^3^ cells per well) were incubated with various concentrations of afatinib and HGF (0, 10, 20, 50 ng/mL) for 72 hours. Cell growth was determined by the MTT assay. The percentage of growth is shown relative to untreated controls. Each sample was assayed in triplicate, with each experiment repeated at least 3 times independently. (TIF) [file pone.0084700.s001.tif]

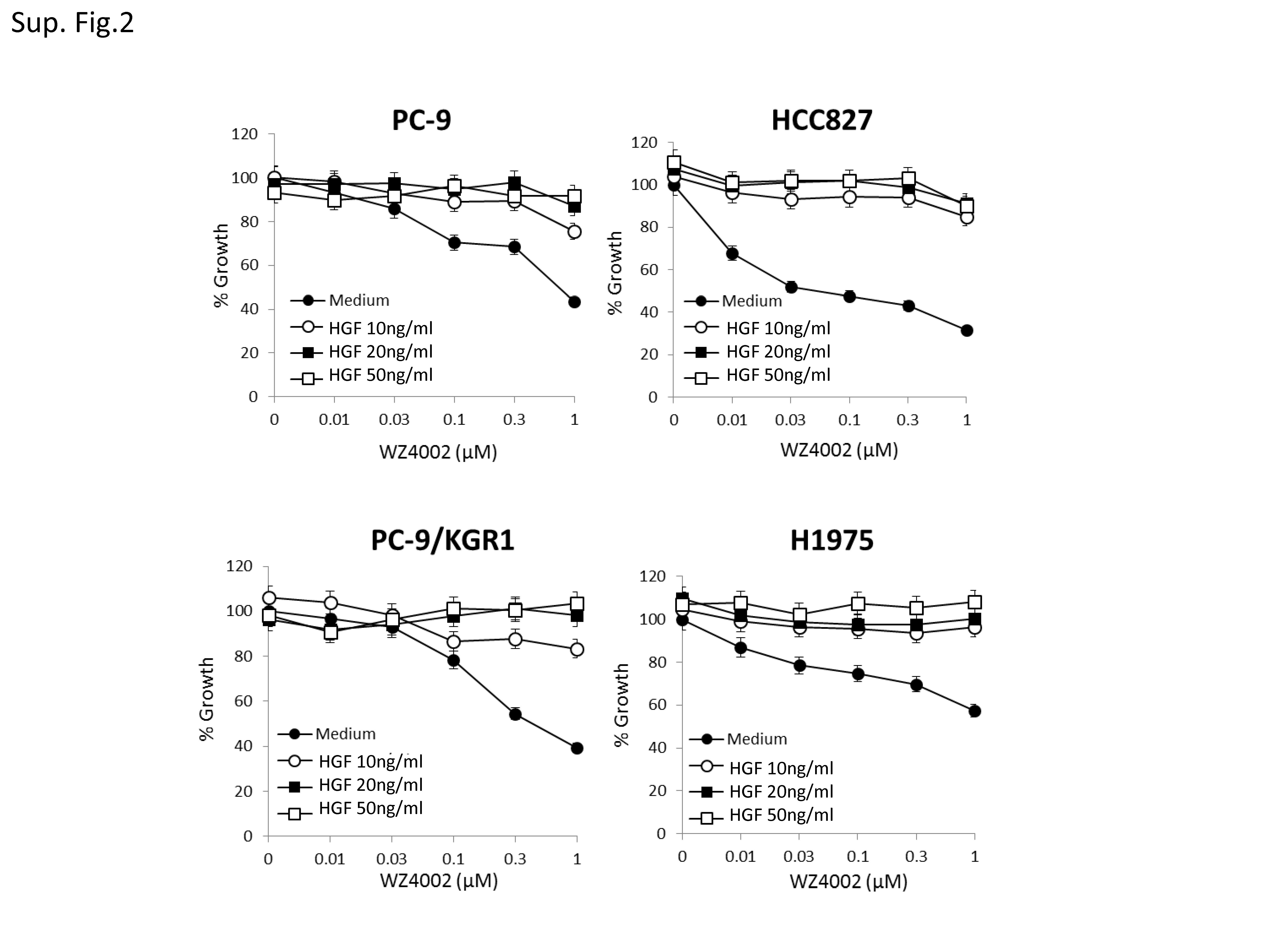

Supplement: Figure S2 — HGF dose-dependently induced resistance to WZ4002 in lung cancer cells harboring EGFR mutations. PC-9, HCC827, PC-9/KGR1, and H1975 cells (2×10^3^ cells per well) were incubated with various concentrations of WZ4002 and HGF (0, 10, 20, 50 ng/mL) for 72 hours. Cell growth was determined by the MTT assay. The percentage of growth is shown relative to untreated controls. Each sample was assayed in triplicate, with each experiment repeated at least 3 times independently. (TIF) [file pone.0084700.s002.tif]

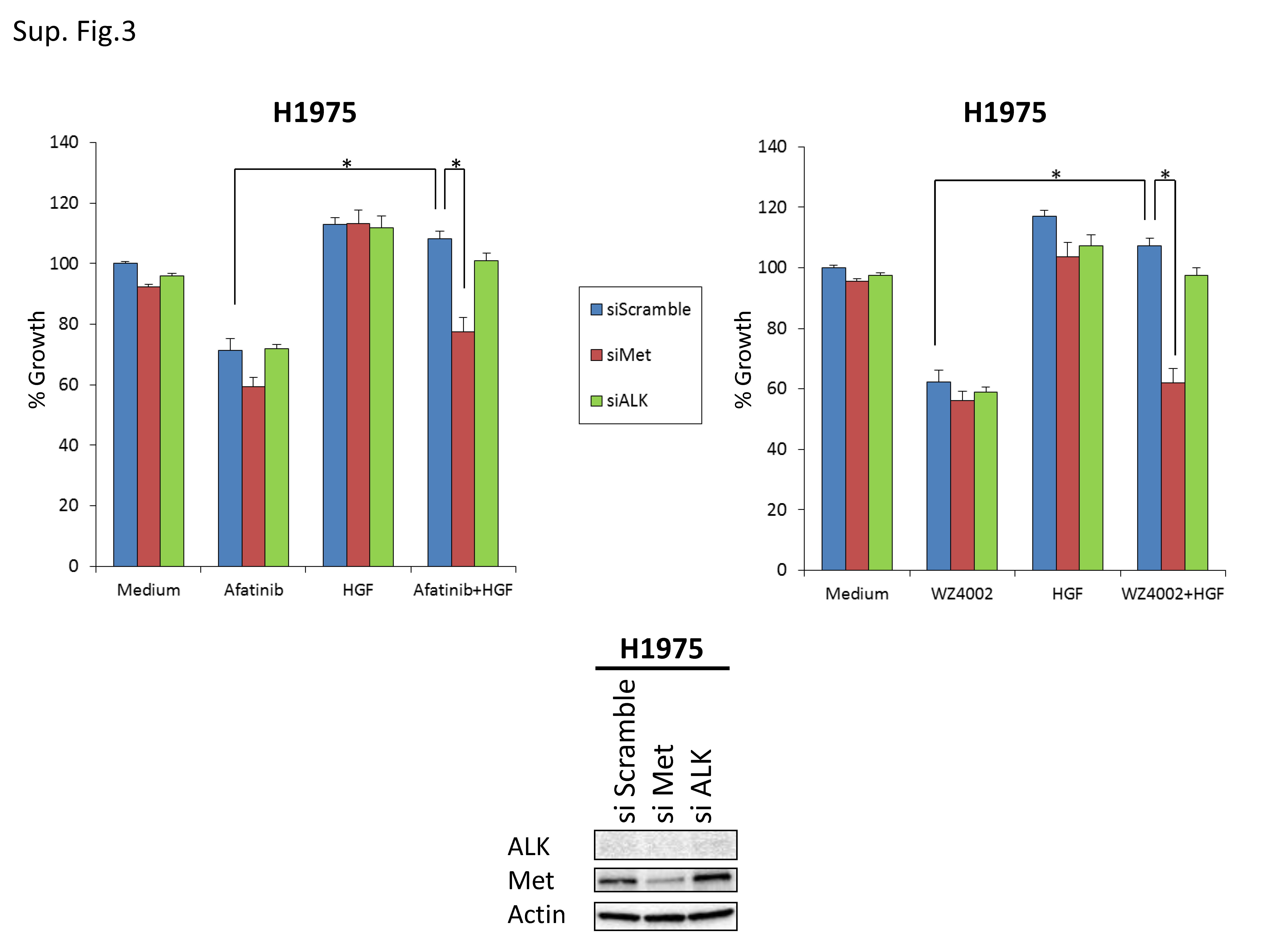

Supplement: Figure S3 — Specific downregulation of Met, but not ALK, reversed afatinib (300nmol/L) or WZ4002 (300nmol/L) resistance induced by HGF (10ng/mL) in H1975 cells. The percentage of growth is shown relative to untreated controls. Each sample was assayed in triplicate, with each experiment repeated at least 3 times independently. *, P < 0.05 by one-way ANOVA. Downregulation of Met or ALK by specific-siRNA was assessed by immunoblotting. (TIF) [file pone.0084700.s003.tif]

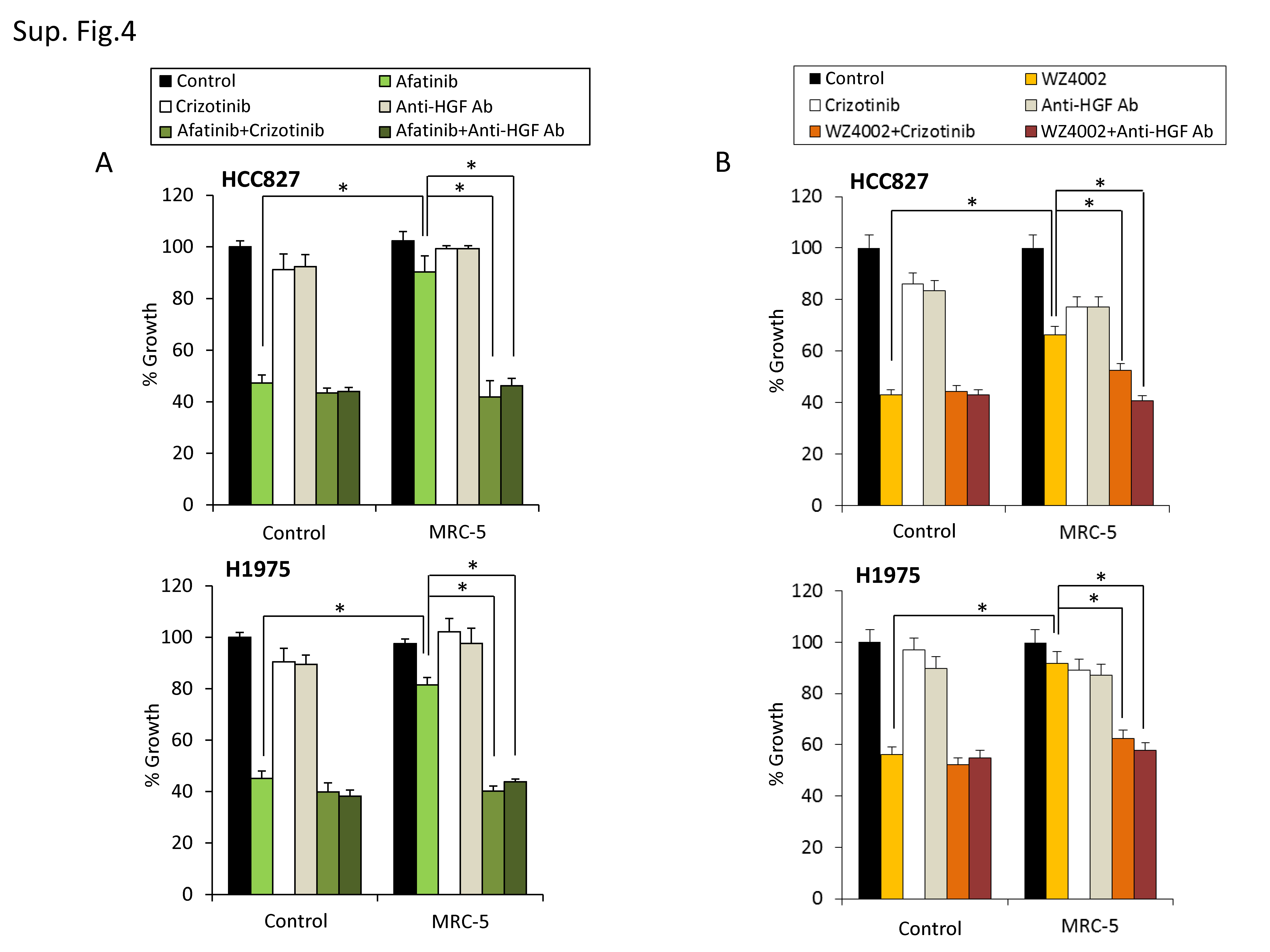

Supplement: Figure S4 — Crizotinib overcomes resistance to new generation EGFR-TKIs caused by fibroblast-derived HGF. Tumor cells (8 × 10^3^ cells/800 μL) were cultured with or without afatinib (100 nmol/L) (A) or WZ4002 (100nmol/L) (B) in the lower chambers of Transwell Collagen-Coated chambers. MRC-5 cells (1 × 10^4^ cells/300 μL), which were or were not pretreated for 2 hours with anti-human HGF antibody (5 μg/mL) or crizotinib (100 nmol/L) were placed in the upper chambers, and the cells were cocultured for 72 hours. The number of cells in the lower chamber was determined by the MTT assay. Percent growth was relative to untreated controls. All samples were assayed at least in triplicate, with each experiment performed three times independently. *, P < 0.05 by one-way ANOVA. (TIF) [file pone.0084700.s004.tif]

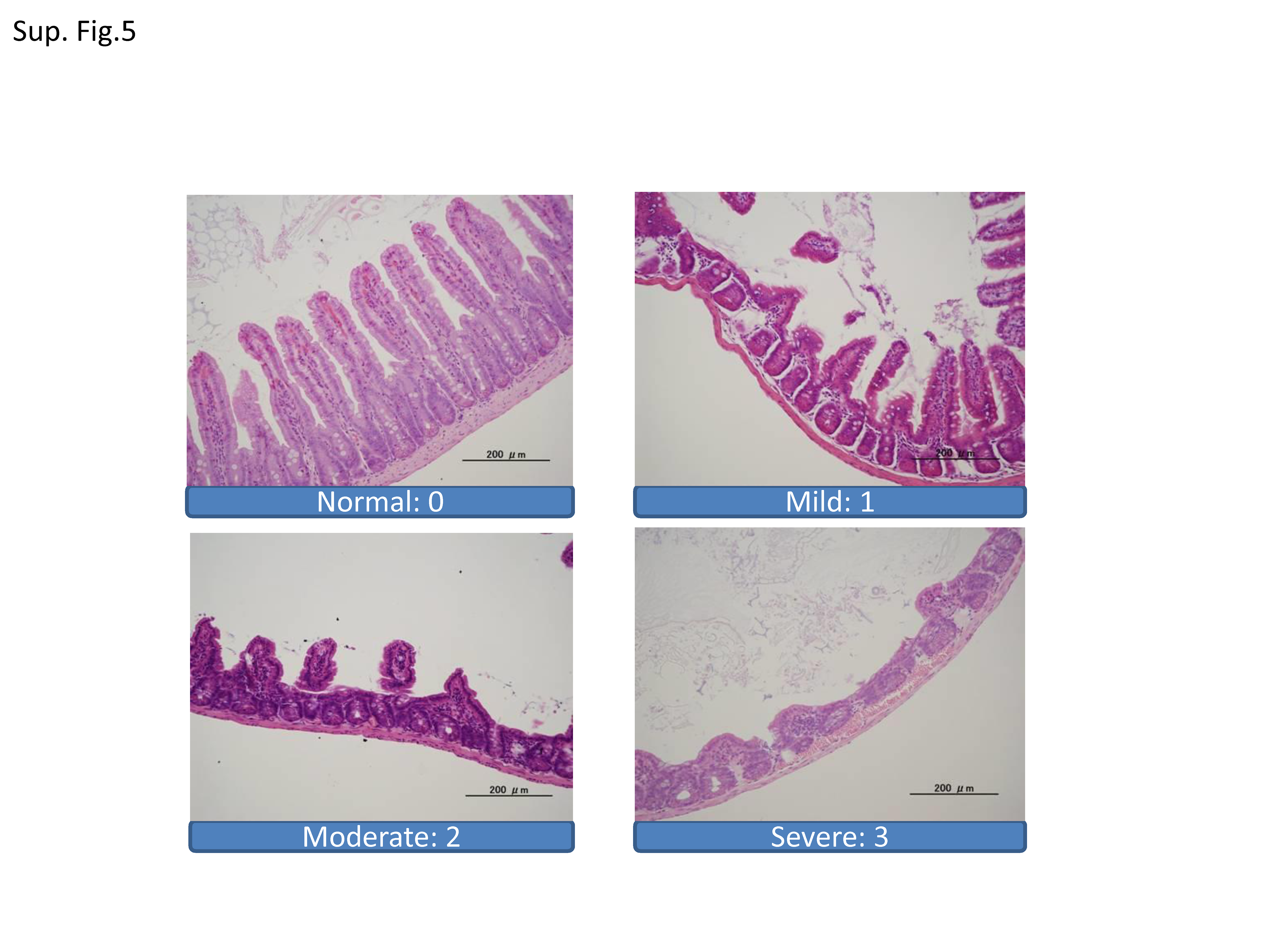

Supplement: Figure S5 — Representative mucosal damage to the small intestine, as assessed by H&E staining. (TIF) [file pone.0084700.s005.tif]

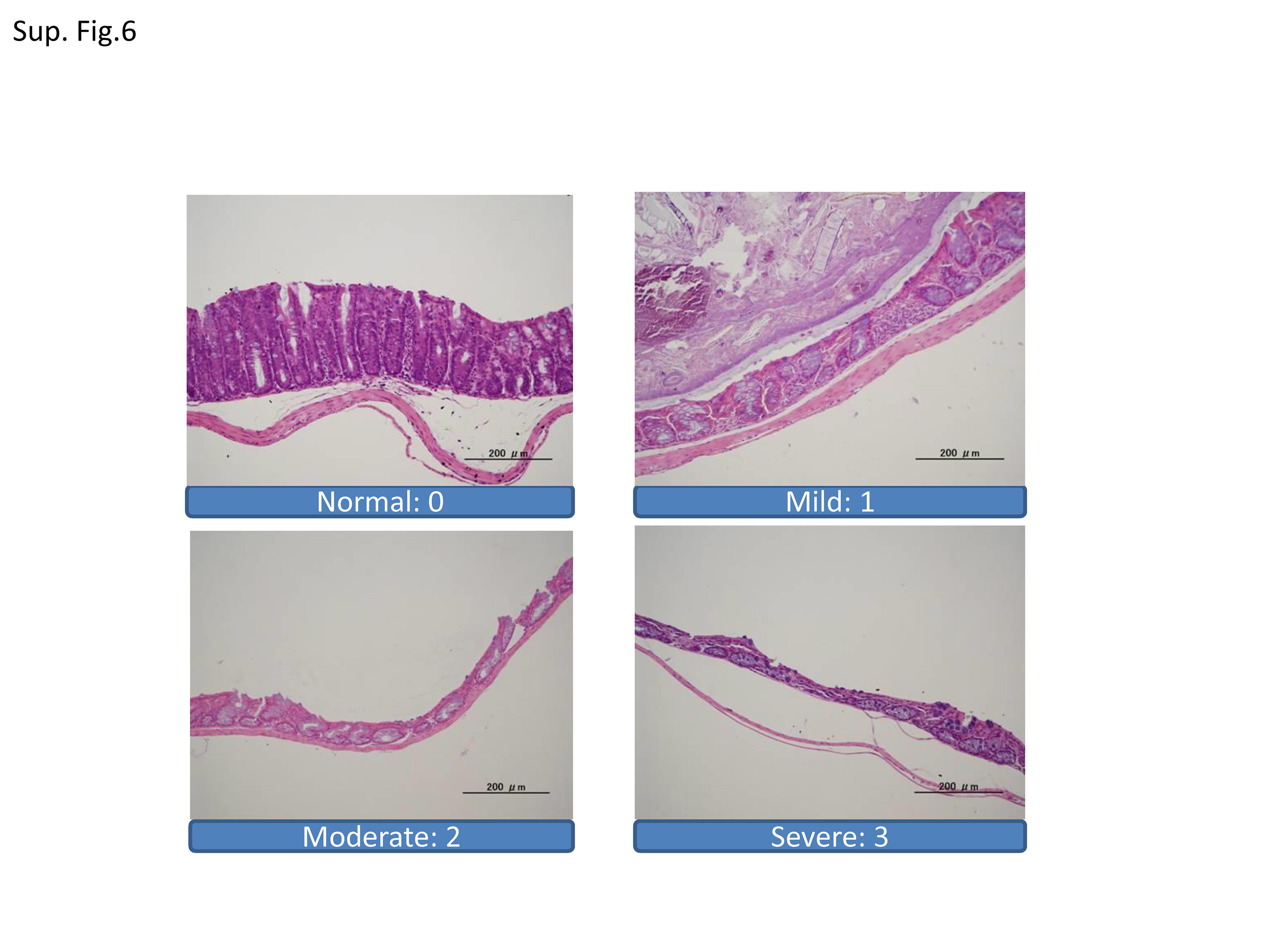

Supplement: Figure S6 — Representative mucosal damage to the large intestine, as assessed by H&E staining. (TIF) [file pone.0084700.s006.tif]
